# Post activation performance enhancement effects on countermovement jumps in men and women

**DOI:** 10.1371/journal.pone.0355738

**Published:** 2026-08-06

**Authors:** Gabriel Felipe Arantes Bertochi, Márcio Fernando Tasinafo Júnior, Leonardo Lima, Kazunori Nosaka, Enrico Fuini Puggina

**Affiliations:** 1 Graduate Program in Rehabilitation and Functional Performance, Ribeirao Preto Medical School, University of Sao Paulo, Ribeirão Preto, São Paulo, Brazil; 2 Graduate Program in Physical Education and Sport, School of Physical Education and Sport of Ribeirao Preto, University of Sao Paulo, Ribeirão Preto, São Paulo, Brazil; 3 School of Medical and Health Sciences, Edith Cowan University, Joondalup, Western Australia, Australia; Eskisehir Technical University, TÜRKIYE

## Abstract

The present study investigated the post-activation performance enhancement (PAPE) effects of six different conditioning activities on countermovement jump (CMJ) height and compared the PAPE effects between sexes. We recruited 18 male and 17 female participants (age: 24.6 ± 3.7 y, height: 1.71 ± 0.10 m, body mass: 75.7 ± 16.1 kg) with at least one year of resistance exercise experience. They performed a familiarization session in which the one-repetition maximum back squat (1-repetition maximum back squat: 134 ± 32 kg; 1- repetition maximum back squat/body mass: 1.8 ± 0.4) was assessed. This was followed by six visits for six different conditioning activities in a randomized order; back squat, concentric-only back squat, eccentric-only back squat, drop jump, drop jump with a weight vest (10% body mass), and eccentric-only drop jump with the weight vest. At each visit, participants performed a standardized warm-up for 10 minutes, followed by three CMJs, the conditioning activity of the day, and after 4 minutes, three CMJs. Thus, post activation performance enhancement was assessed by the change in CMJ height (average of three trials) from pre- to post-conditioning activity. The changes were compared among the six conditioning activities by a 6x2 repeated measures analysis of variance. In the six conditions, no condition x time (pre-post) interaction effect (F_[3.89,34]_=1.71; p = 0.153; ղ^2^_p_=0.048) was evident for all participants, and this was also the case for men (F_[5,17]_=1.54; p = 0.185; ղ^2^_p_=0.083) and women (F_[5,16]_=0.362; p = 0.873; ղ^2^_p_=0.022), without significant differences between sexes. These results showed no PAPE after the conditioning activities within a 4-minute window for moderately trained individuals, and no difference between men and women. It appears that the PAPE effect is small when the conditioning activities are proceeded by warm-up exercise, and no sex difference exists for the effect. In practice, sports and exercise practitioners need to consider the individual characteristics that can influence the PAPE response when isolating the conditioning activity from the warm-up.

## Introduction

Post-activation performance enhancement (PAPE) is the acute improvement in neuromuscular voluntary performance following conditioning activities (CAs) [1]; however, controversy exists regarding which CA leads to a higher PAPE response. Typical CAs include multiple sets of resistance exercises such as back squats (BS) at an intensity of more than 80% of one-repetition maximum (1-RM), or velocity-dependent exercises such as drop jumps (DJs) and loaded DJs [2–4]. CAs focusing on eccentric rather than concentric contractions have been reported to be more efficient in enhancing countermovement jump (CMJ) performance [5,6]. A systematic review demonstrated slightly superior PAPE effect induced by velocity-based exercises such as plyometrics, in comparison to high-intensity resistance exercises [4], which is probably due to less motor pattern interference [1,7]. Additionally, some studies observed significant PAPE represented by an increase in jump height after loaded or eccentric overloaded velocity-based exercises [8–10].

It appears that different forms of CAs, considering the velocity-force spectrum and the phase of the movement (i.e., concentric vs eccentric) affect the magnitude of PAPE. However, no previous study has systematically evaluated the different PAPE responses between concentric or eccentric contractions in the BS and compared them with plyometric activities with or without additional load, to the best of our knowledge. This information can be important in the sports context when considering the best choice of CA to include in a training context like in contrast training.

In many of the previous studies, PAPE was assessed after a general warm-up followed by a CA, and the CMJ height is one of the most described PAPE markers [11]. It has been documented that PAPE can be evoked by a warm-up routine, and its effect is similar to that evoked by a BS or loaded jump [10]. Xu et al. [11] concluded in their review article that when a comprehensive warm-up including all RAMP [raise, activate, mobilize, and potentiate] phases was performed before a CA, the CA produced a trivial or small effect on PAPE. Therefore, if the aim is to investigate the PAPE effects of different CAs, a possible effect of warm-up exercise on PAPE before the CAs should be avoided by inserting sufficient time between them. Accordingly, Chen et al. [12] showed that the time interval after which PAPE effects on jump performance were no longer observed was above 12–15 minutes.

The PAPE effects are modulated across different individual characteristics such as strength level, resistance training experience, and sex [4,11]. However, it is important to note that some controversy exists regarding which sex shows a greater PAPE response. A previous study reported a better response for females than males [11], and a longer lasting PAPE response for males than females [13]. However, Xu et al. [11] stated that the low number of studies investigating PAPE in female participants might generate a potential risk of publication bias. Thus, it is worth investigating the difference in PAPE between men and women with a similar level of training experience and strength. This could help coaches and sports practitioners to understand how effective a CA can be in evoking PAPE according to the sex of the athlete.

Therefore, the present study aimed to investigate the PAPE effects of six different CAs on CMJ height and compare the PAPE effects between males and females. We hypothesized that: (a) the PAPE effects would differ between CAs and velocity-based CAs would show the largest effect; and (b) women would present greater PAPE compared to men. From the present study, it was expected to clarify the PAPE effect regarding different strength-velocity pattern CAs, and potential sex differences in the PAPE, which will provide a better understanding of PAPE for coaches, practitioners, and athletes.

## Materials and methods

### Participants

This study was approved by the Human Research Ethics Committee of the School of Physical Education and Sport of Ribeirão Preto (Ethics Code: 77364724.2.0000.5659). All participants provided written informed consent prior to enrolment in the study. This research was conducted ethically in accordance with the World Medical Association Declaration of Helsinki. Participants were recruited from the university and the community surrounding the university through social media, verbal invitations and flyers. Participants were informed about the benefits, risk and study procedures providing informed consent to participate in the study.

Inclusion criteria were that participants should have at least one year of resistance training experience, not present any musculoskeletal injuries that could interfere with their health or the outcomes of the study, not use illegal ergogenic substances, and be between 18 and 35 years old. Although most of the participants included had a history of resistance training, some participants had a background in individual and team sports. The sample size was calculated for a repeated measures of within-between interaction design. Using an effect size of 0.9 for the BS effect on PAPE from a previous study [14], the sample size was estimated with a power of 0.80, an alpha level of 0.05, and a correlation of 0.5, which indicated that at least 12 participants were necessary. Assuming a possible dropout of 20% among the participants over the study, and doubling the sample for sex comparisons, 35 participants (18 males and 17 females) were recruited. The participants’ characteristics are presented in Table 1.

**Table 1:** Participants’ characteristics.

	Average ± SD (range)	
Characteristics	Males	Females
Age (y)	23.8 ± 4.0 (18–30)	25.5 ± 3.2 (18–30)
Height (m)	1.78 ± 0.06 (1.70–1.90)	1.62 ± 0.05 (1.54–1.75)
Body mass (kg)	84.6 ± 17.4 (63.7–120.3)	66.3 ± 7.1 (58.6–84.4)
BS 1-RM (kg)	149.8 ± 33.7 (90–200)	117.4 ± 20.2 (88–158)
BS 1-RM/BM	1.8 ± 0.4 (1.1–2.7)	1.8 ± 0.3 (1.3–2.5)

### Design and procedures

The present study employed a randomized crossover test-retest design. The recruitment period for participants in this study was from March 11th to July 25th, 2024. Participants were asked to visit the laboratory on seven occasions (one familiarization and six experimental sessions), with at least one day between sessions. The main author conducted the randomization for each participant for the six experimental sessions using a randomization tool (https://www.randomizer.org/). The six experimental sessions were fully randomized without blocking or stratification. Participants were instructed to avoid any strenuous lower body exercise the day before each visit, and to avoid tobacco, alcohol consumption, or stimulant-based supplementation for 12 hours before the visit.

On the first visit, the participants underwent an initial screening to assess the training experience, age, height, body mass, and history of musculoskeletal injury. Afterward, the participants had their back squat (BS) one-repetition maximum (1-RM) assessed and proceeded to the familiarization, to reduce any possible learning and adaptation effects with the countermovement jump (CMJ) and drop jump (DJ) protocols. From the second to the seventh visits, they performed one of the six CAs in a randomized order: eccentric-concentric back-squat (BS), concentric-only back-squat (CON-BS), eccentric-only back-squat (ECC-BS), DJ, DJ + 10% body mass in a weighted vest (Loaded-DJ) and eccentric only DJ + 10% body mass in a weighted vest (ECC-DJ) as explained in detail below. The experimental visits were carried out based on participants’ availability.

All participants performed a standardized warm-up at each experimental condition before the baseline CMJ assessment. The warm-up was structured to obtain the best possible increase in performance with minimal fatigue, based on the RAMP model [15] and previous review suggestions [3,16]. Each participant walked on a treadmill to raise body and muscle temperature (R – raise) and performed body-weight exercises (detailed in the “warm-up exercise” section) to activate and mobilize the muscles involved in CMJ, BS, and DJ (AM – activate and mobilize). The potentiation (P) element was excluded from the warm-up protocol in the present study because this phase consisted of the CA. It has been documented that a warm-up protocol with any of the three elements in RAMP is still considered to be comprehensive [11].

After the warm-up, participants rested for 15 minutes to perform a CMJ test (baseline) followed by another 15-minute rest before performing one of the six different CAs. The 15 minutes period between warm-up and CMJ and CMJ and CA was chosen to avoid any residual PAPE effects from the warm-up or CMJ, as intended in the study aim [12] while ensuring the complete resynthesis of PCr stores [17], and a significantly higher muscle temperature compared to pre-warm-up values, as indicated by previous articles investigating the time course of muscle temperature after warm-up [18]. A second CMJ post-test (post) was performed 4 minutes after the CA. The post-test time period was chosen based on the interval that demonstrates a favored PAPE response for jump (4–7 minutes) based on a recent systematic review with meta-analysis [12]. Each CMJ test was composed of three repetitions with a 15-second rest between repetitions. The mean of the three height measurements was used as the performance outcome since the mean is a more sensible metric to detect neuromuscular status [19].

#### One-repetition maximum (1-RM).

The BS 1-RM test was performed using a Smith machine. Initially, each participant performed two warm-up sets with 20 repetitions (only the bar) and 15 repetitions (10 kg on each side). Then, each participant performed an initial trial with a weight between 50 and 70% of the perceived capacity, with a 10–20% progressive increase in the resistance until the participant could not complete a single repetition [20]. Up to six trials were allowed, with three to five minutes of rest between trials. A bench was positioned just behind the participants’ legs, with its height adjusted to approximately 90 degrees of knee flexion, measured by a manual goniometer, and used with the same individual settings for each participant’s BS CA for standardization.

#### Warm-up exercise.

The warm-up exercise was adapted from the protocol of Piper et al. [21] and composed of five different exercises with a total time of approximately 10 minutes. Participants performed a treadmill walk for five minutes at 4 km/h, followed by walking toe touches, forward lunges, walking quad pulls (each exercise consisting of 2 sets of 5 repetitions for each leg), finishing with bodyweight squats (2 sets of 10 repetitions). The exercises were chosen based on warm-up recommendations, including aerobic exercises, dynamic stretching, and squat-specific exercises [3,16].

#### Countermovement jump (CMJ).

The CMJ test was conducted on an Ergo Jump platform (Cefise®, Nova Odessa – Brazil). The platform determines the jump height through the flight time using the following equation:


$$\begin{array}{c} h=\frac{t^{\text{2}}.g}{\text{8}} \end{array}$$

where h (meters) = jump height; g (meter per second squared) = gravity acceleration; t (seconds) = flight time [22].

Each participant was positioned on the platform, standing with their hands on the waist. Afterward, they were instructed to jump as high as possible and land with the knees straight. Three attempts were allowed with 15-second rest intervals [23]. The coefficient of variation based on the three baseline CMJs for each participant was 3.2 ± 2.0% with the ICC being 0.993, and 95% CI being 0.992 to 0.995.

#### Perceived readiness.

Participants were asked to rate the perceived readiness scale [24] after each CA, immediately before performing the post-CMJ test. The perceived readiness scale is based on seven items from 1: “fully recovered” to 7: “exhausted” to self-determine readiness for the CMJ test. Participants indicated one number on the scale that most represented their perceived readiness right before the CMJ test. The perceived readiness analysis was conducted with 34 individuals, as data were missing for one female participant.

### Conditioning activities

#### Back squat.

Participants performed three BS CAs (traditional concentric-eccentric [BS], eccentric-only [ECC-BS], and concentric-only [CON-BS]) on a smith machine. For BS, three sets of five repetitions, with an 87% of 1RM and a three-minute rest interval between sets were allowed [21]. BS protocols were matched for the number of contractions [25]; thus, ECC-BS and CON-BS were performed with three sets of ten repetitions with 87% of 1RM and a three-minute rest between sets.

#### Concentric-eccentric, concentric-only, and eccentric-only BS.

The BS protocol involved both concentric and eccentric phases of BS, whereas the ECC-BS protocol involved only the downward movement in BS, and the CON-BS only the upward movement in BS. To perform the CON-BS and ECC-BS conditions, the participants were asked to start with a standing position, with the bar near the participant’s trapezius muscles while two researchers were positioned at each side of the bar. For CON-BS, the participant was asked to sit while the researchers held the bar and performed the downward movement, gently letting the bar on the participant’s trapezius muscles, followed by the upward movement performed by the participant. The ECC-BS condition was performed similarly to the CON-BS, but the participant started performing the eccentric phase with the researchers lifting the bar after the participant reached the 90º knee joint angle followed by the participant standing without extra load, preparing for the next repetition.

#### Drop jump.

Participants were positioned on a 40 cm platform, which is the closest height a previous study indicated as the optimal height for DJ [10]. They were instructed to step off the platform with one foot, drop from the platform, and land on the ground with both feet simultaneously with a subsequent jump as high as possible. They were advised to maintain their hands on their hips during the DJs. Verbal encouragement was provided during all trials. Three sets of five repetitions were performed [23,26,27] with a three-minute rest between sets.

A pilot study with three males (height: 1.77 ± 0.05 m; body mass: 75.1 ± 17.4 kg; age: 27.3 ± 1.5 y; BS 1-RM: 127.7 ± 62.7 kg) and two females (height: 1.66 ± 0.04 m; body mass: 62.9 ± 4.7 kg; age: 26.0 ± 1.4 y; BS 1-RM: 108.0 ± 28.3 kg) demonstrated a larger effect size for PAPE in CMJ height when using a weighted vested of 10% than a 20% body mass. Therefore, each participant used a trunk-weighted vest with 10% body mass for the loaded-DJ and ECC-DJ. The loaded-DJ was performed in the same way as that described above. In the ECC-DJ, participants did not perform the subsequent jump, performing only the step-off and landing phase of the DJ. Participants performed three sets of 10 repetitions for ECC-DJ to match the number of contractions with the others DJ protocols.

### Statistical analyses

Statistical analyses were assessed in JAMOVI v. 2.6.44. The Shapiro-Wilk test was used to assess normality. No violation of normality was observed in the baseline CMJ considering the six conditions. The normality was rejected for the BS, CON-BS, and ECC-BS conditions in the whole data, considering the comparison among six CAs for all participants (n = 35). For men, the normality was rejected for the BS only, but no violation of normality was found for women. When the normality was rejected, the data were log-transformed for statistical analyses.

The variation between the baseline CMJ measurements was quantified by coefficient of variation and intraclass correlation coefficient with a 95% confidence interval. The ICC was calculated based on a two-way mixed model with absolute agreement type. To check the order effect of the six CA conditions, one-way repeated measures ANOVA was used to compare the first to sixth sessions in the order that each CA was performed for each individual considering the baseline CMJ height. A 6 x 2 (condition x time) repeated measure ANOVA was used to compare between the six CA conditions for the changes in CMJ height before and after the CA for all participants together (n = 35). A similar analysis was performed for male (n = 18) and female participants (n = 17), separately.

To examine individual responses, we investigated the number of participants who showed an increase in CMJ height of more than 3.2% was counted for each CA. The one-way repeated measures ANOVA and the 6 x 2 (6 conditions, 2 time points) repeated measures ANOVA for all participants violated the assumption of sphericity, therefore a Greenhouse-Geisser correction was applied. When a condition x time significance was observed, the Bonferroni post-hoc was performed. Partial eta squared values of ≤ 0.01, ≤ 0.06, and ≥ 0.14 were considered small, medium, and large effect sizes, respectively [28,29]. A repeated measures Friedman test with Durbin-Conover post-hoc evaluated the differences between the median of perceived readiness ratings among each CA condition. A 5% alpha level for statistical significance was set. Data was reported as means ± standard deviations (SD) and [range] for CMJ height and % change, and as median and interquartile range (25^th^ – 75^th^ quartile) for Perceived readiness ratings.

## Results

**Fig 1:**
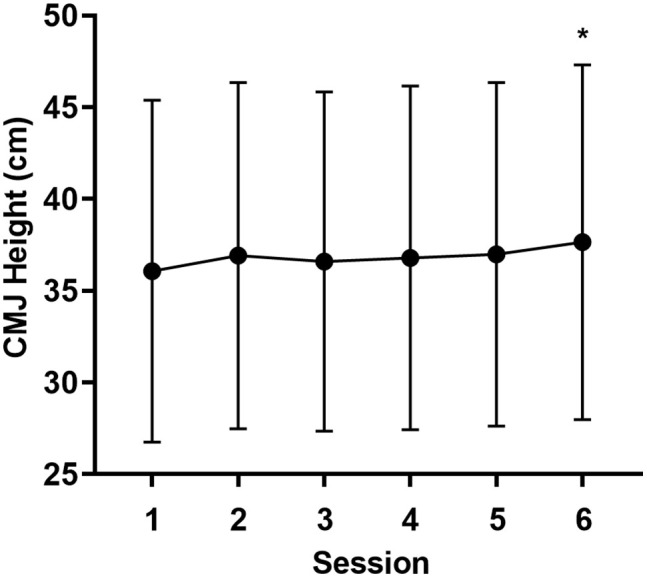
Raw countermovement jump height (cm) data at baseline over seven sessions. *Significantly different from 1^st^, and 3^rd^ sessions.

As shown in Fig 1, a significant increase in the baseline CMJ height was evident over the six sessions (F_[3.57,34]_ = 4.00; p = 0.006; ղ^2^_p_ = 0.105 [medium]; є = 0.714). Post-hoc analysis demonstrated a significantly higher CMJ in the sixth session when compared with the first (4.4%) and third (2.9%) sessions.

### Perceived readiness

**Fig 2:**
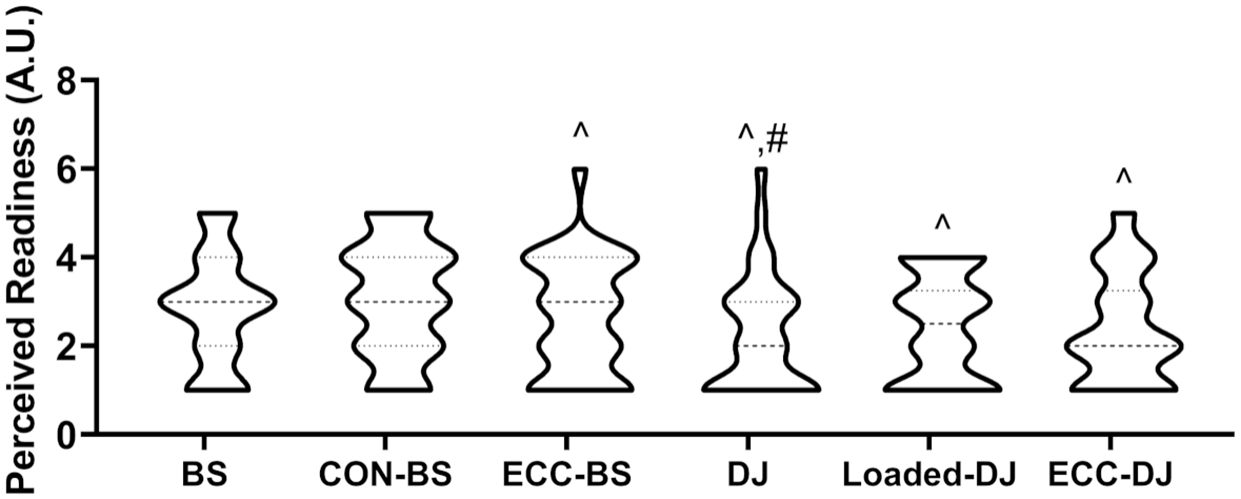
Perceived readiness rating after back squat (BS), concentric-only BS (CON-BS), eccentric-only BS (ECC-BS), DJ, DJ with weight vest (Loaded-DJ), and eccentric-only DJ (ECC-DJ) conditioning activities. The darker dotted line represents the mean, while the dotted lines below and above represent the interquartile range (25th and 75th, respectively). The values of 34 participants are shown. #: significantly (p < 0.05) different from BS; ^: significantly (p < 0.05) different from CON-BS.

The perceived readiness ratings significantly differed between CAs (X^2^ = 15.9; p < 0.01) as shown in Fig 2. The perceived readiness was significantly higher (p < 0.05) after BS (3 [2–3.75]) when compared to DJ (2 [1 –3]), and after CON-BS (3 [2 –4]) compared to ECC-BS (3 [1.25–4]), DJ (2 [1 –3]), loaded-DJ (2.5 [1 –3]), and ECC-DJ (2 [1 –3]).

### Comparison among six CAs for all participants

**Fig 3:**
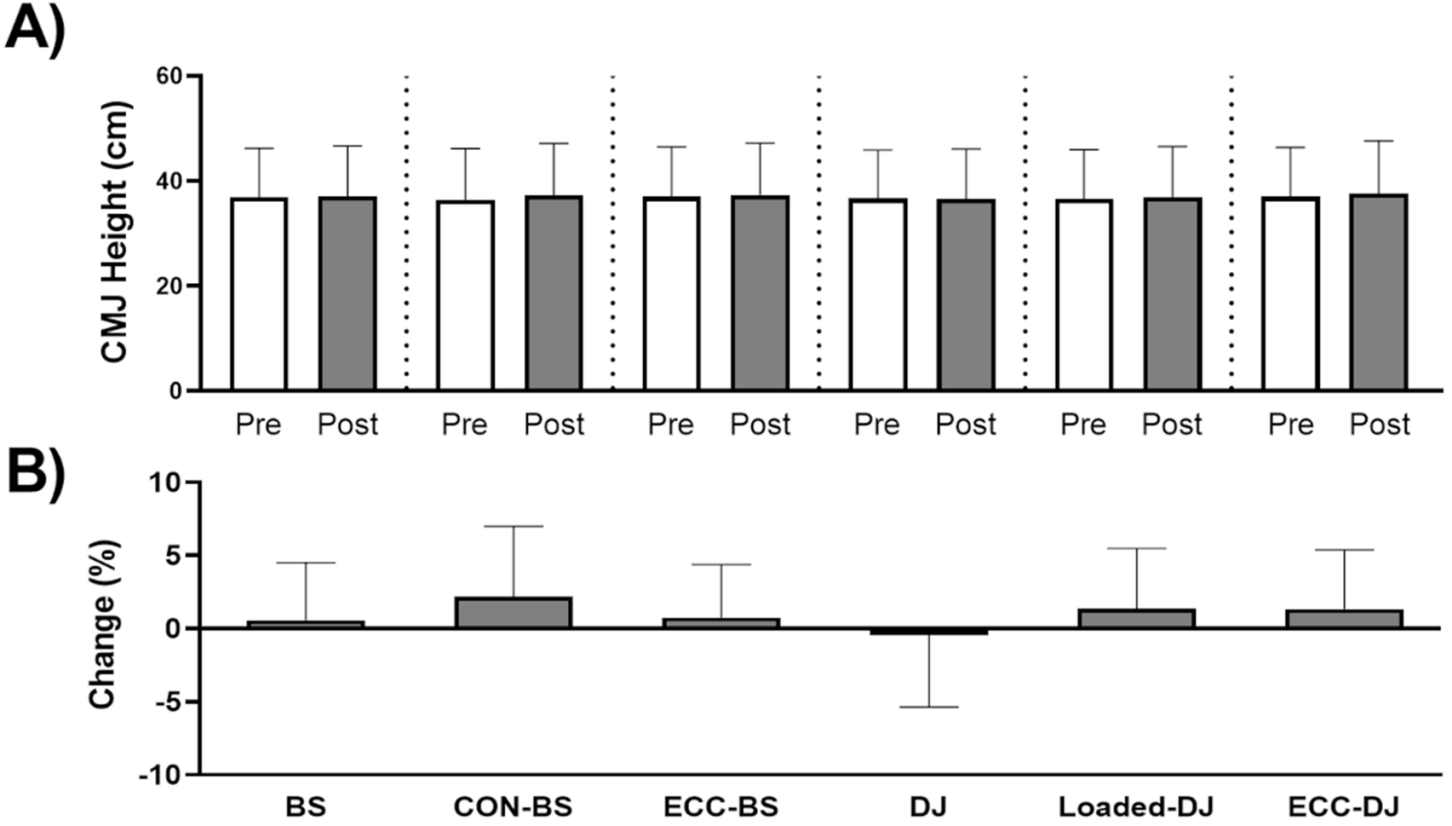
Raw countermovement jump height data before (pre) and after (post) each conditioning activity; back squat (BS), concentric-only BS (CON-BS), eccentric-only BS (ECC-BS), DJ, DJ with weight vest (Loaded-DJ), and eccentric-only DJ (ECC-DJ) [A] and percentage change in the height from pre- and post-intervention [B]. The mean and SD values of 35 participants are shown.

Fig 3 shows changes in CMJ jump height before (pre) and after (post) each CA, and the percentage change in CMJ jump height for each CA. When comparing the six conditions, no condition x time (pre-post) interaction effect (F_[3.89,34]_ = 1.71; p = 0.153; ղ^2^_p_ = 0.048 [medium]; є = 0.778) was evident.

When examining the individual responses to each CA based on an increase of more than 3.2% in CMJ height from pre- to post-CA, no participant presented an increase for all CAs together. However, eight participants presented an increase for BS (5.8%), 13 for CON-BS (7.1%), eight for ECC-BS (5.4%), six for DJ (8.0%), 11 for Loaded-DJ (6.3%), and 12 for ECC-DJ (5.6%). The maximum number of CA that a single person showed an increase above 3.2% was five (n = 1).

### Male vs female participants

**Fig 4:**
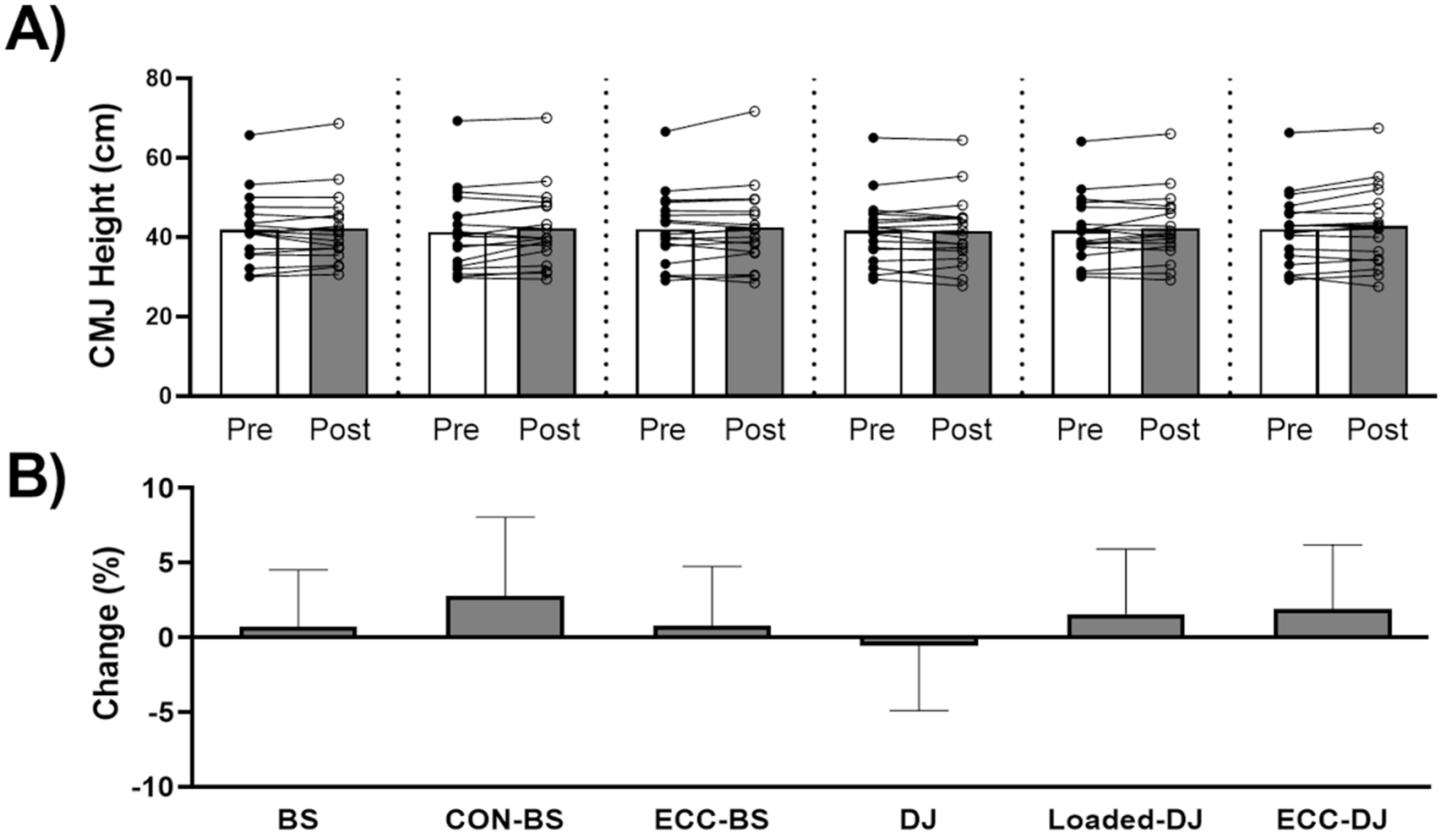
Raw countermovement jump height data before (pre) and after (post) each conditioning activity; back squat (BS), concentric-only BS (CON-BS), eccentric-only BS (ECC-BS), drop jump (DJ), drop jump with weight vest (Loaded-DJ), and eccentric-only DJ (ECC-DJ) for men [A] and percentage change in the height from pre- and post-intervention [B]. The mean and SD values of 18 men and their individual responses are shown.

**Fig 5:**
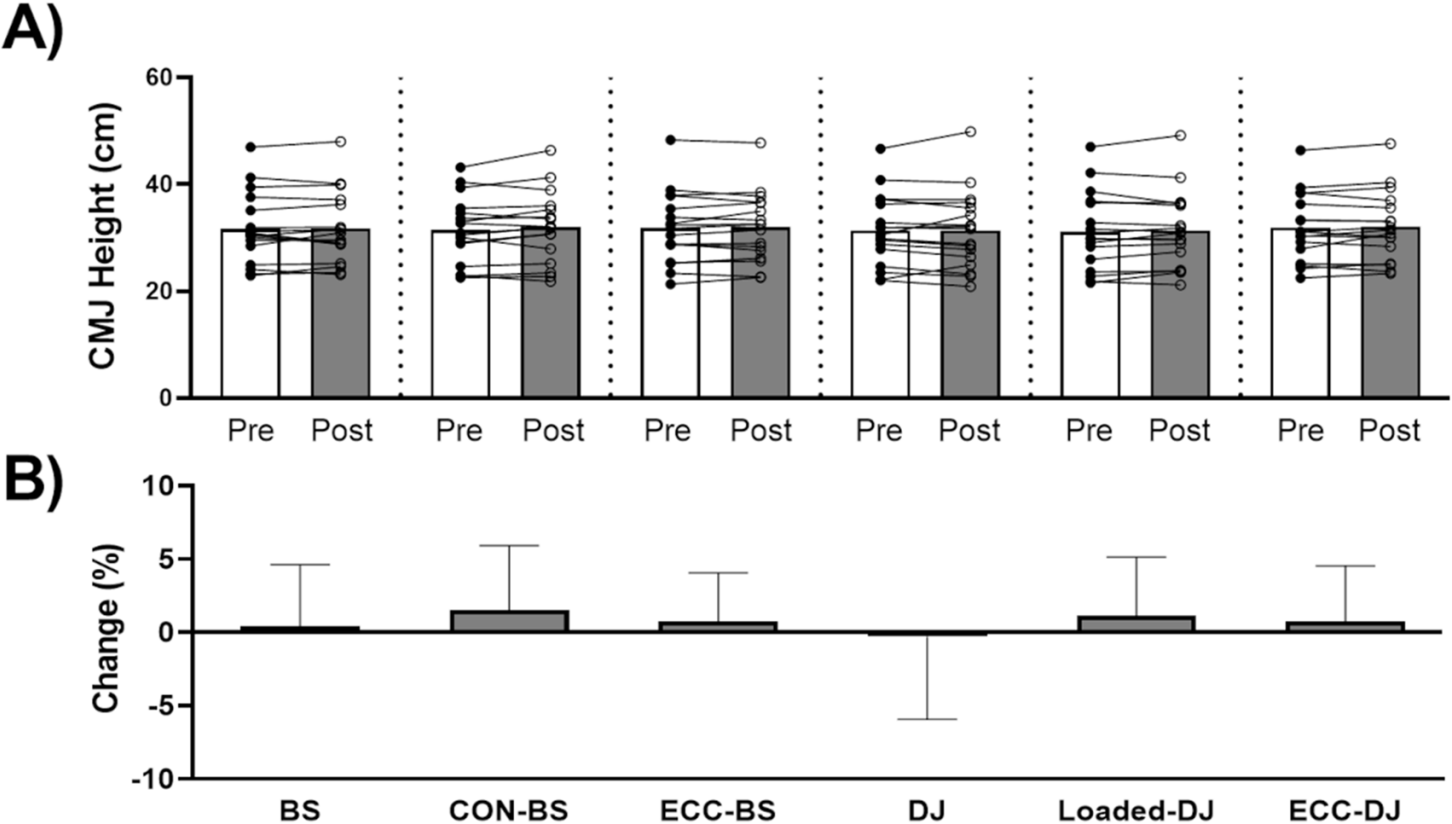
Raw countermovement jump height data before (pre) and after (post) each conditioning activity; back squat (BS), concentric-only BS (CON-BS), eccentric-only BS (ECC-BS), drop jump (DJ), drop jump with weight vest (Loaded-DJ), and eccentric-only DJ (ECC-DJ) for women [A] and percentage change in the height from pre- and post-intervention [B]. The mean and SD values of 17 women and their individual responses are shown.

When comparing males and females for their responses, neither men (F_[5,17]_ = 1.54; p = 0.185; ղ^2^_p_ = 0.083 [medium]; є = 0.679; Fig 4) nor women (F_[5.16]_ = 0.362; p = 0.873; ղ^2^_p_ = 0.022 [medium]; є = 0.717; Fig 5) presented any condition x time interaction effect. Regarding the individual responses, five male participants presented increases in CMJ height above the measurement error (3.2%) after BS (5.1%), seven after CON-BS (8.1%), three after ECC-DJ (6.6%), three after DJ (5.5%), six after Loaded-DJ (6.5%), and eight after ECC-DJ (5.7%). The maximum number of CA that a single male showed an increase above 3.2% was five by one participant. For female, three participants presented an increase in CMJ height after BS (6.9%), six after CON-BS (6.0%), five after ECC-BS (4.7%), three after DJ (10.5%), five after Loaded-DJ (6.2%), and four after ECC-DJ (5.4%). The maximum number of CA that a single female showed an increase above 3.2% was three by four participants.

## Discussion

The results showed that: 1) no significant PAPE was found after any CA (Fig 3); and 2) there were no differences in the PAPE response between males and females (Figs 4 and 5). These results did not support the hypothesis that: (1) the PAPE effects would be different depending on the CA and velocity-based CAs would show the largest effect; and (2) women would present greater PAPE compared to men after different CAs.

Before discussing the results further, the study design of the present study requires attention. In the present study, the same participants performed six different CAs across six sessions. As shown in Fig 1, a significant increase in the baseline CMJ height was observed over six sessions, more precisely from the first to the sixth (4.4%) as well as the third to the sixth session (2.9%). Considering the magnitude of noise for the CMJ height measure identified in the present study (3.2%), these increases were modest but still relevant. Thus, the increase observed across sessions in baseline CMJ can indicate a potential learning effect. Given that the PAPE effect is generally small (around 4–5%) [10], it is possible that the learning effect affected PAPE responsiveness. Nonetheless, it is important to note that the order of the six CAs was randomized among the participants, and no significant difference was evident for the baseline CMJ height among the conditions (Fig 3). Thus, it seems that the study design was still adequate to compare different CAs for their PAPE effects on CMJ height.

In the present study, no CMJ height measure was taken before the initial warm-up exercise, so it is not known whether CMJ height increased immediately after the initial warm-up exercise. Not many previous PAPE studies measured the effects of the warm-up exercise performed before a CA on PAPE. However, those that did it reported different changes in CMJ height, ranging from −2.4% [30] to 7.0% [31]. For example, Fletcher [31] analyzed 16 male collegiate athletes and found that the increase in CMJ height after a warm-up exercise consisting of aerobic and dynamic stretching exercises was 7% when CMJ height was measured 4 minutes after the warm-up exercise. It should be noted that the baseline CMJ height measure was performed 15 minutes after the warm-up exercise in the present study. The muscle temperature after warm-up exercise gradually decreases, with a major decrease after 10 minutes [32]. However, Tsurubami et al. [18] showed that there was still a significant increased muscle temperature at 20 minutes after the warm-up compared to the baseline, although the effect depended on the intensity of the warm-up. Thus, this aligns with our goal of isolating the PAPE warm-up effects from the CA, while still maintaining a higher muscle temperature compared to before the warm-up, although we cannot confirm this based on our data.

To the best of our knowledge, no previous study has used the 15-minute and four-minute time frame between the initial warm-up exercise and baseline CMJ, and between a CA and post-CA assessment, respectively. However, two studies [10,33] had a similar time frame between the initial warm-up exercise and baseline CMJ measure to that of the present study. Filip-Stachnik et al. [33] evaluated the CMJ height at 2, 4, 6, 8, and 10 minutes after BS. The authors reported no PAPE for any of the time points. However, it should be noted that Filip-Stachnik et al. [33] evaluated only females for their responses to BS and stated that the reasons for the lack of PAPE might be due to the different muscle fiber proportion, muscle strength between males and females, and training experience among the participants. Additionally, having more training experience (e.g., more familiarized) with the CAs exercise may enable individuals to perform more work with less fatigue [7]. Although the participants in the present study had at least one year of resistance exercise training experience, many were not familiar with plyometric exercises.

Saez Saez de Villarreal et al. [10] examined the effects of different CAs (warm-up, DJ, BS, loaded-jump, and no-CA) on CMJ performed five minutes after the CAs, and reported a small but significant increase in CMJ height after warm-up (7.0%), BS (4.6–5.0%), and loaded jump (4.1%). They stated that the PAPE was induced by increased motor neuron excitability after heavy loading exercise and an increase in muscle temperature after warm-up. However, the load in BS used in the study by Saez Saez de Villarreal et al. [10] would allow participants to perform more repetitions than expected compared to the load used in the present study. For example, based on the relationship between % 1-RM and the number of repetitions, up to eight repetitions would be possible for the 80% 1-RM load, but in the study of Saez Saez de Villarreal et al. [10] the participants performed only four repetitions. In the present study, 87% of 1-RM load was used, and the participants performed five repetitions (maximum number of repetitions for the load, based on the relationship between % 1-RM and repetitions). It may be that more fatigue might have been induced in the present study. Since the PAPE response to a CA is a net balance between fatigue and potentiation [4], no increases in CMJ height after the six CAs may be associated with a more fatigued state, indicated by a perceived readiness rating different from one (fully recovered) across the six CAs (Fig 2), and the lack of training experience in the participants in the present study.

Our results demonstrated that some participants were PAPE responders to different CA (participants that presented an increase in CMJ above our error measurement) and others were not considered PAPE responders. Mola et al. [34] analyzed the PAPE responses at 15 sec, 4, 8, 12, 16, and 20 minutes after BS for PAPE responders versus non-responders based on an increase of CMJ height more than 1.6 cm after the exercise. They reported no PAPE in CMJ height for PAPE non-responders, whereas a significant increase in CMJ was observed for PAPE responders at 16 minutes after the exercise. The authors suggest that PAPE responders present greater BS strength (96.7 kg) than non-responders (80.0 kg) explaining the large PAPE response. Seitz and Haff [4] stated that that stronger individuals in BS responded better to PAPE. Therefore, a better PAPE response can be expected for stronger individuals considering movements with similar muscle groups. In the present study, the mean relative 1-RM BS/BM considering the 35 participants was 1.8. Therefore, although our sample is considered stronger based on previous research [4] this was not sufficient to elicit PAPE in any CA. This reinforces the need for PAPE strategies to be individualized, since factors other than strength, such as, training experience, age, and fiber type [35] may contribute to the PAPE response.

When comparing male and female participants (Figs 4 and 5), there were no differences observed between them. Sex has previously been considered an influencing factor for PAPE and warm-up responses [13,36]. Some studies have compared men and women for their responses to CAs with conflicting results [13,36]. Herring et al. [36] found no PAPE irrespective of the sex at 4–7 minutes after plyometrics or midthigh isometric pulls. Koźlenia and Domaradzki [13], found a significant increase in CMJ at 3 minutes after isometric BS for both sexes (4.5% for men, 6.5% for women), but the PAPE effect lasted until 9 minutes after the exercise for men only, when the PAPE was assessed at 1, 3, 5, 7 and 9 minutes post-exercise. It has been stated that women are less responsive to PAPE because of the lack of strength and less muscle mass when compared to men [13,37]. However, the mean relative strength of the male and female participants was not largely different in the present study (1.81 kg/kg for men, and 1.78 kg/kg for women). For this condition, it does not appear that sex differences existed.

### Limitations

One of the limitations of the present study was that the experimental visits were not necessarily conducted at the same time of day or with consistent daily intervals. Furthermore, there was no monitoring of the participants’ nutrition, hydration status and caffeine consumption. Additionally, the significant differences observed in baseline CMJ over the six sessions because of the high number of experimental sessions may have induced training adaptations that influenced fatigue recovery and, consequently, responsiveness to PAPE. Moreover, only a 4-minute window PAPE was captured, which may have limited a full PAPE time course analysis, some participants may exhibit delayed potentiation peaks (above 4 minutes). Nonetheless, although the sample size meets the previously calculated number of participants, we acknowledge that an effect size of 0.9 is considered large, which may compromise the sensitivity of the study to detect smaller PAPE effects. Finally, the present study evaluated moderately trained individuals, thus, the conclusions should not be extrapolated to more highly trained individuals.

## Conclusions

The present study showed that when the CA is isolated from the warm-up PAPE effects no PAPE was observed from different CA on a 4-minute window for moderately trained individuals. Nonetheless, it is important to note that some participants showed a relatively larger PAPE than others. In some cases, the magnitude of the increase in CMJ height was large (>10%), which can be individually influenced by training experience, age, and fiber type [35]. This should not be ignored, but the reasons for the individual differences were beyond the scope of the present study. In practice, sports and exercise practitioners need to consider the individual characteristics that can influence PAPE response when isolating the CA from the warm-up.

## Supporting information

S1 File PAPE data. (XLSX)
